# Association of a Specific *OsCULLIN3c* Haplotype with Salt Stress Responses in Local Thai Rice

**DOI:** 10.3390/ijms25021040

**Published:** 2024-01-15

**Authors:** Bagus Herwibawa, Chakkree Lekklar, Supachitra Chadchawan, Teerapong Buaboocha

**Affiliations:** 1 Program in Biotechnology, Faculty of Science, Chulalongkorn University, Bangkok 10330, Thailand; bagus.herwibawa@live.com; 2 Biological Sciences Program, Faculty of Science, Chulalongkorn University, Bangkok 10330, Thailand; l.chakkree@gmail.com; 3 Center of Excellence in Environment and Plant Physiology, Department of Botany, Faculty of Science, Chulalongkorn University, Bangkok 10330, Thailand; supachitra.c@chula.ac.th; 4 Omics Science and Bioinformatics Center, Faculty of Science, Chulalongkorn University, Bangkok 10330, Thailand; 5 Center of Excellence in Molecular Crop, Department of Biochemistry, Faculty of Science, Chulalongkorn University, Bangkok 10330, Thailand

**Keywords:** CRE, cullin, indel, phylogenetic, SNP, TFBS

## Abstract

We previously found that *OsCUL3c* is involved in the salt stress response. However, there are no definitive reports on the diversity of *OsCUL3c* in local Thai rice. In this study, we showed that the *CUL3* group was clearly separated from the other CUL groups; next, we focused on *OsCUL3c*, the third *CUL3* of the *CUL3* family in rice, which is absent in Arabidopsis. A total of 111 SNPs and 28 indels over the *OsCUL3c* region, representing 79 haplotypes (haps), were found. Haplotyping revealed that group I (hap A and hap C) and group II (hap B1 and hap D) were different mutated variants, which showed their association with phenotypes under salt stress. These results were supported by cis-regulatory elements (CREs) and transcription factor binding sites (TFBSs) analyses. We found that *LTR*, *MYC*, [*AP2*; *ERF*], and *NF*-*YB*, which are related to salt stress, drought stress, and the response to abscisic acid (ABA), have distinct positions and numbers in the haplotypes of group I and group II. An RNA Seq analysis of the two predominant haplotypes from each group showed that the *OsCUL3c* expression of the group I representative was upregulated and that of group II was downregulated, which was confirmed by RT-qPCR. Promoter changes might affect the transcriptional responses to salt stress, leading to different regulatory mechanisms for the expression of different haplotypes. We speculate that *OsCUL3c* influences the regulation of salt-related responses, and haplotype variations play a role in this regulation.

## 1. Introduction

The ten largest rice-producing countries are China, India, Indonesia, Bangladesh, Vietnam, Thailand, Myanmar, the Philippines, Japan, and Brazil [1]. Five of these countries are in Southeast Asia, which has long been producing yields of rice that exceed the regional demand and thus are exported to other countries [2]. Thailand is one of the largest rice-exporting countries, and its production has increased by 64.83% in the last 40 years [3]. Thus, Thailand has many opportunities in the future, mainly because global agriculture must increase its food supply by 56% to feed the growing population. Considering climate change, this estimate could reach 62% [4]. Sudden climate change caused by natural and anthropogenic factors has occurred in the last two decades, intensifying the threat of biotic and abiotic disturbances to crops. These global changes include a rise in sea level, which increases the salinity of surface water and groundwater through the intrusion of salt water [5]. It has been reported that the rise in the water surface affects salt intrusion in the Chao Phraya River, affecting the water quality in Bangkok, Samut Prakan, and Nonthaburi [6].

The seawater intrusion on agricultural soil properties in Nonthaburi increased the electric conductivity (EC) and sodium adsorption ratio (SAR) to approximately 0.21–4.38 dS m^−1^ and 8.29–41.89, respectively, which are higher than the annual average value [7]. Moreover, in the northeast of Thailand, approximately 17% of its land area is affected by salt from rock salt and the accumulated effects from human activities [8]. The effect of high salinity is continuing to expand in some regions, thus potentially threatening rice production in Thailand. Therefore, we must explore the genetic resources of rice to understand the underlying mechanism in its response to salt stress. Salt stress is an ionic imbalance because of the excessive accumulation of Na^+^ and Cl^−^, thus disturbing the metabolism of other essential ions; a concentration of NaCl above 30 mM (EC~3 dS m^−1^) can decrease the production of modern rice by 12% per dS m^−1^ and the EC at 6 dS m^−1^ can decrease production by up to 50%. The selection of rice germplasm to identify genes related to salt tolerance has been previously carried out using genome [9], transcriptome comparison, and through combining genome and gene co-expression networks [10].

Moreover, a meta-analysis successfully detected 9366 salt-tolerant candidate genes. Interestingly, 81 genes are on chromosome 1; chromosome 12 has the lowest number of genes (14 genes) and chromosome 8 has the highest number of genes (868 genes) [11]. Among the candidate genes on chromosome 8 is a gene from *cullin* family based on a genome-wide association study (GWAS) in 104 local Thai rice: *OsCUL3c* (*Os08g0170900*) [12]. However, the role of *OsCUL3c* in rice remains unclear. A BLAST search against the National Center for Biotechnology Information (NCBI) GenBank database revealed that *OsCUL3c* is only found in rice. *OsCUL3c* can be differentiated from other rice *CUL3s*, such as *OsCUL3a* and *OsCUL3b* [13,14,15]. *OsCUL3a* has a role in negatively regulating cell death and immunity [16,17,18], while *OsCUL3b* influences the crown root number [15]. Rice CUL3s, together with the broad complex/tramtrack/bric-a-brac and ring-finger protein ring box protein 1 (CUL3-BTB-RBX) complex, is one of the four subtypes of cullin–RING ligases (CRLs) [19]. In a previous report, the presence and absence of variations (PAV), possibly caused by retrotransposon movement, may impact *OsCUL3c* expression and plant height phenotype [20].

CRLs are ubiquitin ligase enzymes (E3), which have many sub-types [21]. E3 ligases use cullins as a scaffold to form multisubunit E3 complexes, and then the cullins bind a target recognition subcomplex and the RBX1 docking protein, which delivers the activated ubiquitin. In plants, E3 is a single protein subunit or multi-subunit protein complex which performs a ubiquitination function via the ubiquitin–proteasome system (UPS) [22]. The UPS plays a role in decreasing the critical component of stress signaling [23]. UPS activity increases during oxidative stress because of the accumulation of damaged proteins [24]. Oxidative stress is caused by the excessive production of reactive oxygen species (ROS) that must be scavenged through detoxification as a strategy to protect against salt stress. The difference in UPS activities can also be viewed as one of the results of the variation in *OsCUL3c*, which can play an essential role in the response to salt stress. However, the association between haplotype variation on the *OsCUL3c* region and salt tolerance has never been confirmed. Moreover, Thailand has abundant local rice germplasm that has not yet been explored. This study used genome sequencing data to perform a haplotype-based association analysis. A total of 233 local Thai rice accessions were randomly selected as haplotype representatives in validation of their association with salt stress responses in rice under salt stress.

## 2. Results

### 2.1. Phylogenetic Analyses of the Cullins Family in Arabidopsis and Rice

**Figure 1:**
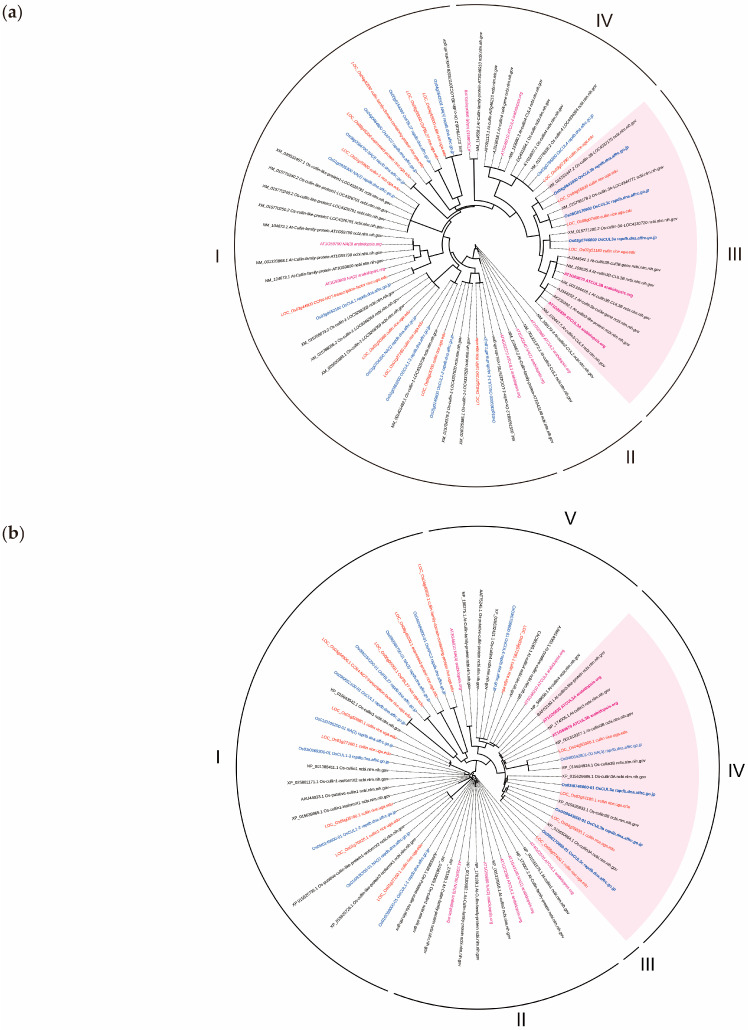
Phylogenetic trees of the *cullin* family in *Arabidopsis thaliana* and *Oryza sativa*. (**a**) Phylogenetic analysis of 71 *cullin* genes and (**b**) 65 cullin proteins. The blue, purple, red, and black letters indicate the data retrieved from the RAP-DB, TAIR, MSU-RGAP, and NCBI repositories, respectively (accessed on 24 March 2023). The pink rectangle highlights the CUL3 family groups in both species.

Since the cullins family (CULs) in both Arabidopsis (*Arabidopsis thaliana*) and rice (*Oryza sativa* L.) data in public databases is not well annotated as a whole, we constructed phylogenetic trees based on DNA (71 members) and protein (65 members) sequences to investigate the relationship between these sequences and to group similar and homologous sequences. The model finder we used tested up to 286 DNA models and 546 proteins to increase the accuracy. The DNA-based phylogenetic tree constructed from 9 Arabidopsis Information Resource (TAIR), 14 MSU Rice Genome Annotation Project (MSU-RGAP), 14 Rice Annotation Project Database (RAP-DB), and 34 NCBI sequences was distributed among four clades (Figure 1a). Each clade showed a grouping based on each *CUL* type. This suggests that the separation of *CUL* genes from the four clades occurred earlier than the speciation of Arabidopsis and rice. Among them, clade I contained the group annotated as *CUL1* in Arabidopsis and rice; however, there were other annotations, such as *OsAPC2* and *OsFBL27* from RAP-DB and *OsCUL3b* from NCBI with ambiguous annotations because they are not consistent with other sources. While clade II contained the group annotated as *CUL2* that is only found in Arabidopsis, we have yet to see this annotation in rice from all the repositories we checked. Clades III and IV contained groups annotated as *CUL3* and *CUL4*, respectively.

The protein-based phylogenetic tree constructed from 9 TAIR, 14 MSU-RGAP, 14 RAP-DB, and 28 NCBI sequences (Figure 1b) contained five clades. Slightly different from the previous results, clade I contained the group annotated as rice CUL1. Other annotations were also found, such as OsAPC2 and OsFBL27. Clade II contained the group annotated as Arabidopsis CUL2. Clade III contained the group annotated as Arabidopsis CUL1. Clades IV and V contained the group annotated as CUL3 and CUL4, respectively. Based on the DNA- and protein-based phylogenetic trees, we can observe that the CUL family sequences for Arabidopsis and rice present in public repositories have yet to be well annotated, especially the CUL1 and CUL2 groups. However, we can conclude that the CUL3 group is separated from the other CUL groups.

### 2.2. The Conservation of Gene Structure and Motifs among CUL3s in Arabidopsis and Rice

**Figure 2:**
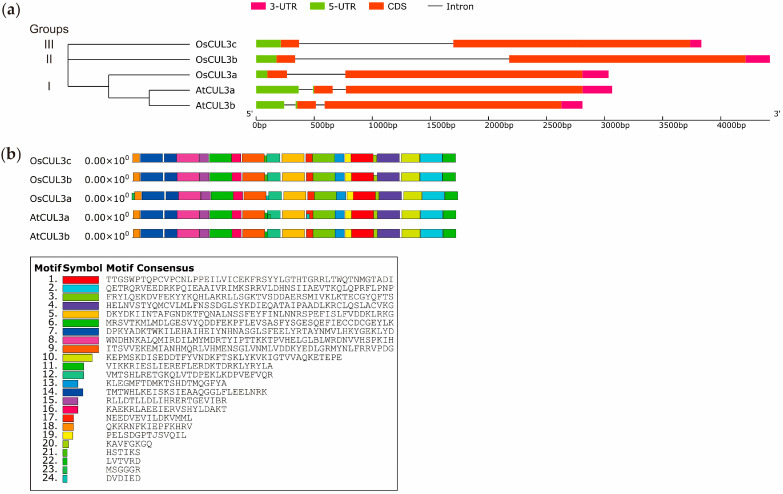
(**a**) Phylogenetic tree of the *CUL3* genes from *Arabidopsis thaliana* and *Oryza sativa*, and (**b**) conserved motifs in CUL3 proteins from *Arabidopsis thaliana* and *Oryza sativa*. The red, green, and purple boxes represent the coding sequence (CDS), 5′- and 3′-untranslated regions (UTRs), respectively. The black line represents introns. Different conserved motifs are displayed as different-colored boxes.

Subsequently, we focused on the *CUL3s* as there are at least two genes in Arabidopsis and rice, *CUL3a* and *CUL3b*, while rice has a third gene, *CUL3c*. A phylogenetic tree was constructed and was divided into three groups (Figure 2a). Group I consisted of *AtCUL3a*, *AtCUL3b*, and *OsCUL3a*, while groups II and III consisted of *OsCUL3b* and *OsCUL3c*, respectively. In both species, the lengths of the coding sequences (CDSs) were identical. However, the structures showed that *CUL3s* contain two introns in Arabidopsis, one in the 5′ untranslated region (5′ UTR), and one intron in rice. The position of the introns in the coding sequence is highly conserved and among them, with the *OsCUL3b* intron being the longest. This comparative analysis revealed the presence of *CUL3s* from the same group that share different gene structures, even though the CDS lengths are conserved.

Subsequently, we examined the predicted motif of the CUL3s using MEME. The smaller the *p*-value, the more significant the match. In total, 24 motifs were revealed, and each CUL3 contained 21 to 23 motifs (Figure 2b). Similar to the gene structure, the CUL3s members from the same group shared different motif compositions. Some motifs were present in specific proteins, implying the functional diversity of the CUL3s. For example, Motif 20 is identical in all members of group I, but different in size from groups II and III; Motifs 22 and 23 are present exclusively in the AtCUL3a and OsCUL3a, respectively, suggesting that they might be linked to functional specificity among these groups of proteins. Next, we focused on OsCUL3c as the third CUL3 of the CUL3 family in rice, which is not present in Arabidopsis.

### 2.3. Allele Variation and Haplotype Analysis of OsCUL3c

**Figure 3:**
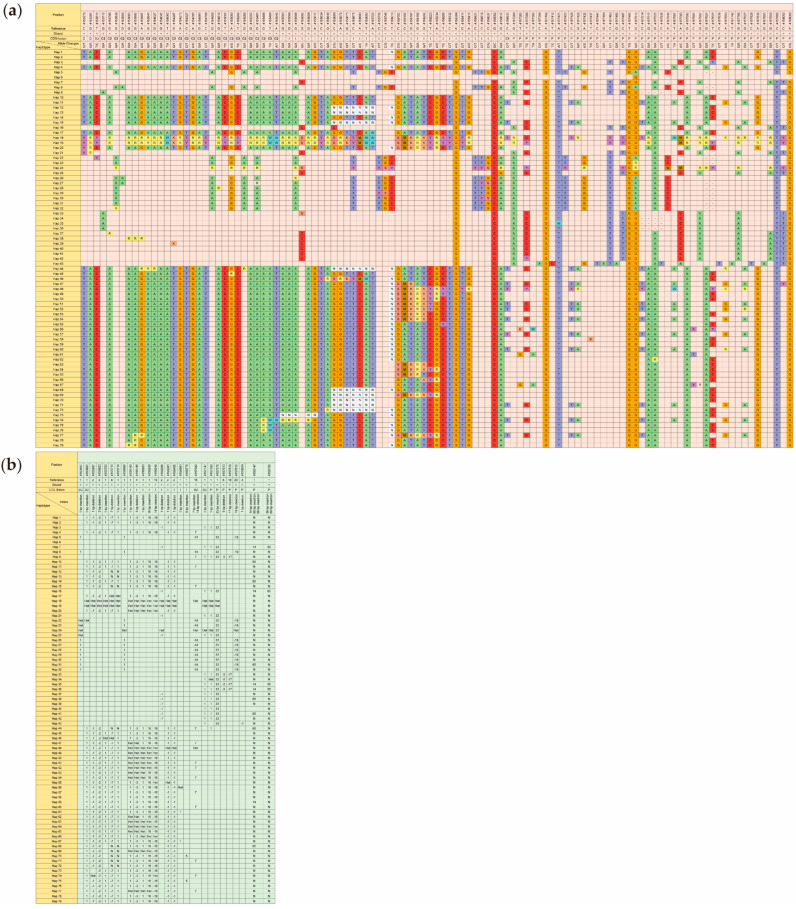
(**a**) Single-nucleotide polymorphism (SNP)- and (**b**) insertion/deletion (Indel)-based haplotype analysis of *OsCUL3c* in 233 accessions of local Thai rice. Light-orange-colored columns provide the list of haplotypes. Light-red-colored columns indicate all SNP variations. Light-green-colored columns represent variation sites for indels. Blank cells indicate reference alleles in those positions. A dash (-) indicates the position of an “unknown” nucleotide or generally refers to “N” (any nucleotide A, T, G, or C). Ambiguity codes W (A or T), S (G or C), M (A or C), K (G or T), R (A or G), and Y (C or T) represent positions when there is some uncertainty between two nucleotides. “Het” refers to “heterozygote”. “P, C, I, 5U, and 3U” in the CDS/intron bar refer to “promoter, coding sequences, intron, 5′- and the 3′-untranslated region”, respectively.

According to the reference genome, there was a total of 111 SNPs in *OsCUL3c* from 233 local Thai rice accessions (Table S1). We found 44 SNPs in the promoter region, 35 SNPs in the intron, 29 SNPs in the CDS, 1 SNP in the 3′-UTR, and 2 SNPs in the downstream sequence (Figure 3a). Based on the nucleotide substitutions, the detected SNPs were classified as transitions (C/T and G/A) and transversions (G/T, C/G, A/T, A/C). Transitions and transversions accounted for 75.68% (84) and 24.32% (27), respectively, with a ratio of 3.11. In the transitions, the number of C/T was lower than that of A/G, which totaled 32 (38.10%) and 52 (61.90%), respectively. For the transversions, the numbers of the four types are slightly different. The numbers of G/T, C/G, A/T, and A/C accounted for 25.93% (7), 22.22% (6), 33.33% (9), and 18.52% (5), respectively. We also observed 28 indels, including 8 indels in the promoter, 2 indels in the 5′-untranslated regions, 16 indels in the intron, and 2 indels in the 3′ untranslated regions (Figure 3b). It was obvious that mononucleotide indels were the most abundant type, accounting for 53.57% (15) of the total indel sites. At the same time, most non-repeat indels were 2 to 66 bp in length, with 10 insertion and 9 deletion types.

Using these 139 SNP and indel data, 79 distinct haplotypes (hap) were obtained (Figure 3a,b). We found that the number of SNPs in each haplotype ranged from 1 to 82, with the minimum number of SNPs in hap 6 and the maximum number in hap 18. At the same time, the number of SNPs for each haplotype in the CDS ranged from 0 to 25, with the minimum number of SNPs in hap 3, hap 6, hap 7, hap 16, hap 21, hap 25, hap 40, hap 41, hap 42, and hap 43, and the maximum number in hap 44. There were 10 haplotypes with no SNPs in the CDS, indicating that the haplotype variations are present in the introns, promoters, or UTRs. In addition, it also explains how the proteins from these haplotypes have the same characteristics as the reference protein. In all haplotypes, there were no C/G or A/C transversion in the CDS. It seems that G/T and A/T transversions are also very uncommon, with the maximum numbers being 1 and 3, respectively. In contrast, the C/T and G/A transitions showed higher values with the maximum numbers being 5 and 15, respectively.

We also noted that some of the differences among haplotypes are due to indels. The presence of indels is also often viewed as a potential source of triplet instability. We determined the indels distribution and revealed that the number in each haplotype ranged from 0 to 18, with the minimum number found in hap 6 and the maximum number in hap 18. Together with the SNP results, we can conclude that hap 6 is very similar to the reference, while hap 18 is the most different when compared to the reference. We did not examine indels in depth because we did not find them in the CDS, and they might not significantly affect regulation. Nevertheless, we needed to consider the indels in the promoter. The promoter determines the response factors, which ultimately affect gene expression. Eight indels were identified in the promoter with a range of 1 to 66 bp; however, indels were not found in 43 haplotypes (54.43%).

**Figure 4:**
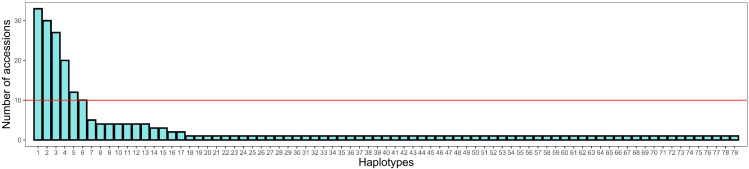
*OsCUL3c* haplotype frequency in 233 accessions of the local Thai rice collection (Table S2). The solid red line indicates the threshold number of accessions.

To further explore the roles of haplotypes in regulation related to salt stress, we selected some reliable main haplotypes. Among the 233 local Thai rice accessions included in this study, the highest frequency was for hap 1, which consisted of 33 accessions, while hap 2, hap 3, hap 4, hap 5, hap 6, and hap 7 consisted of 30, 27, 20, 12, 10, and 5 accessions, respectively. Hap 8 to hap 13 consisted of four accessions. Hap 14 and hap 15 consisted of three accessions. Hap 16 and hap 17 consisted of two accessions. Hap 18 to hap 79 were each represented by only one accession. We realize that most existing haplotypes are unreliable because many have only one accession. Therefore, we set a threshold value of 10 accessions per haplotype. For further insight, we specified only six main haplotypes, i.e., hap 1, hap 2, hap 3, hap 4, hap 5, and hap 6 (Figure 4).

**Figure 5:**
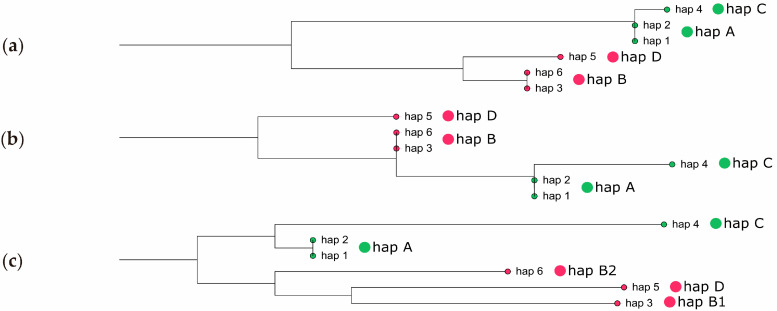
(**a**) Phylogenetic trees of coding sequences-based haplotypes, (**b**) amino acid-based haplotypes, and (**c**) promoter-based haplotypes.

The alignment of six main haplotypes based on coding sequences (CDSs) with 27 allele changes is shown in Figure S1. As expected, most of the allele changes were silent mutations as the amino acid (AA)-based haplotypes showed four residue changes (Figure S2). From the CDS and AA phylogenetic trees, we generated four new haplotype groups, i.e., hap A consisting of hap 1 and hap 2, hap B consisting of hap 3 and hap 6, hap C consisting of hap 4, and hap D consisting of hap 5 (Figure 5a,b). We can see the difference more clearly in the alignment of the six main haplotype promoters (Figure S3). The promoter phylogenetic tree showed slightly different haplotype groups; i.e., hap A consisted of hap 1 and hap 2, hap B1 consisted of hap 3, hap B2 consisted of hap 6, hap C consisted of hap 4, and hap D consisted of hap 5 (Figure 5c).

### 2.4. CREs and TFBSs in OsCUL3c Putative Promoter

**Figure 6:**
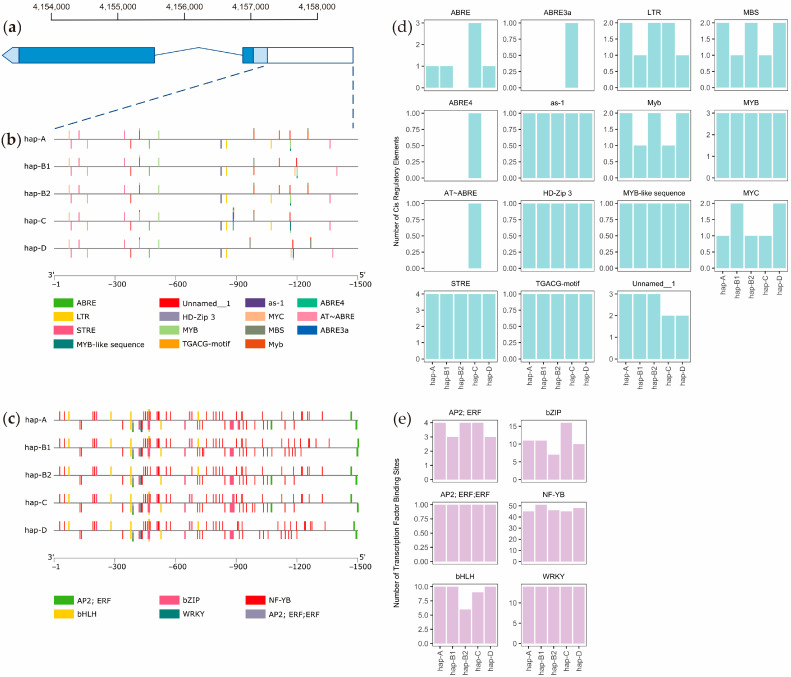
(**a**) Gene structure of *OsCUL3c* with the putative promoter region. The light blue, grey, and white boxes represent coding sequences (CDSs), untranslated regions (UTRs), and putative promoters, respectively. Solid blue lines represent introns. (**b**) The predicted cis-regulatory element (CRE) and (**c**) transcription factor binding site (TFBS) positions in the putative promoter sequences. The putative CREs and TFBSs at certain positions are illustrated by distinct colored rectangles. (**d**) The number of CREs and (**e**) TFBSs. Data are only shown for response factors related to salt and drought stress and the response to abscisic acid (ABA).

The alignment of predominant haplotypes based on their promoters showed several variants (SNPs and indels) that might contribute to gene regulation. To identify the cis-regulatory elements (CREs) using PlantCARE and transcription factor binding sites (TFBSs) using PlantPan in the promoter, the 1500 bp upstream region upstream of the ATG was scanned (Figure 6a). Our analysis revealed 792, 813, 836, 782, and 797 binding sites that were found on hap A, hap B1, hap B2, hap C, and hap D from both databases, with minimum and maximum lengths of 4 bp and 21 bp, respectively. The CREs consisted of 38 elements, including salt- and drought-stress responsive (*as-1*, *HD-Zip 3*, *LTR*, *MBS*, *Myb*, *MYB*, *MYB-like sequence*, *MYC*, *STRE*, *TGACG*-*motif*), light-responsive (*ACE*, *ARE*, *box 4*, *G-box*, *LAMP element*, *MRE*, *sp1*, *TCCC motif*, *TCT motif*), anoxic-inducibility (*GC motif*), wounding- and pathogen-responsive (*WRE3*, *WUN motif*), abscisic acid-responsive (*ABRE*, *ABRE3a*, *ABRE4*, *Unnamed__1*), auxin-responsive (*TGA* element), methyl jasmonate-responsive (*CGTCA motif*), cellular development (*Unnamed__4*), core (*CAAT box*, *AT~ABRE*, *AT~TATA box*, *TATA box*) and unknown (*A*-*box*, *CCGTCC motif*, *CCGTCC box*, *Unnamed__2*, *motif_sequence*) (Figure S4a) elements. Signature response factors were detected: *ABRE3a*, *ABRE4*, *ACE*, and *AT~ABRE* were specific for hap C; *ABRE* was not found in hap B2; and *LTR* and *MYC* were differentially found between hap A/C and hap B1/D.

The TFBSs consisted of 33 factors, including salt-stress-responsive (*bHLH*, *bZIP*, *WRKY*), dehydration-responsive ([*AP2*; *ERF*], [*AP2*; *ERF*; *ERF*]), iron-responsive (*B3*), ethylene-responsive (*AP2*, *ERF*, *EIN3*, [*AP2*; *B3*; *RAV*]), auxin-responsive ([*B3*; *ARF*], [*B3*; *ARF*; *ARF*]), abscisic-acid-responsive (*NF-YB*), gibberellin-responsive ([*Myb*/*SANT*; *MYB*], *MADF*, *Myb*/*SANT*), growth and development (*TCR*, *SBP*, [*NAC*; *NAM*], *alpha-amylase*, *LEA_5*), and unknown (*TBP*, *TCP*, *AT-Hook*, *GATA*, [*Dof*; *GATA*], *LOB*, *Homeodomain*, [*Homeodomain*; *ZF-HD*], [*Homeodomain*; *HD-ZIP*], [*Homeodomain*; *TALE*], others, motif sequence only) factors (Figure S4b). Signature response factors were detected: *ERF* was not found in hap B1, and *MADF* was not found in hap A and hap D. To understand the relationships between the response factors and haplotype, we identified the variations in the positions of the response factors related to salt and drought stress and the response to abscisic acid (ABA), i.e., *LTR*, *MYC*, [*AP2*; *ERF*], and *NF-YB*, that have specific positions and numbers for group I (hap A and C) and group II (hap B1 and D) (Figure 6b–e). Moreover, *WRE-3* is related to wounding and pathogen response, [*Myb*/*SANT*; *MYB*] is related to the gibberellin response, *LOB* and [*Homeodomain*; *TALE*] have unknown functions; these also have specific positions and numbers for each of the both haplotype groups (Figure S4a,b).

### 2.5. Salt Tolerance Index in Local Thai Rice Accessions

**Figure 7:**
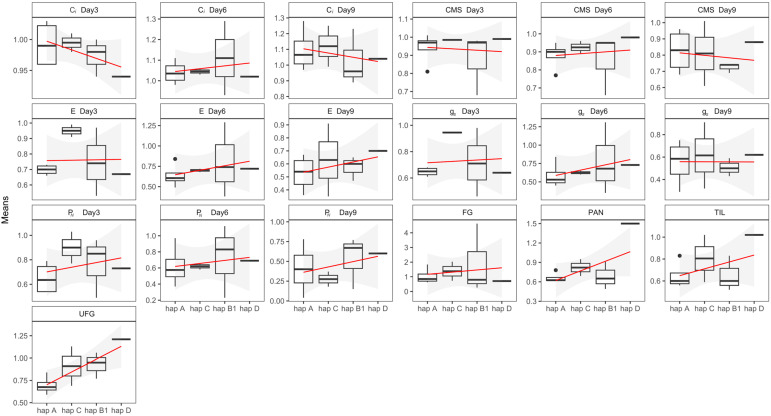
Association of predominant *OsCUL3c* haplotypes and nineteen stress susceptibility indices (SSI) for salt tolerance-related traits. All phenotypes measured were evaluated under 150 mM NaCl for 3, 6, and 9 days [12]. The red line represents the linear line of best fit between the haplotypes and SSIs. C*_i_*, intercellular CO_2_ concentration; CMS, cell membrane stability; E, transpiration rate; g*_s_*, stomatal conductance; P*_N_*, net photosynthetic rate; FG, number of filled grains; PAN, number of panicles; TIL, number of tillers; UFG, number of unfilled grains.

We used the phenotype data provided by Lekklar [12] (Figure S5) to analyze the associations between the stress susceptibility indices (SSI) of these phenotype parameters and the predominant haplotypes (group I (hap A and hap C) and group II (hap B1 and hap D)) (Figure 7). We used Pearson correlation to perform linear regression analyses of the haplotypes for each parameter. The results revealed that almost all parameters had the same trend in their response to salt stress. For C*_i_* Day3, C*_i_* Day9, CMS Day3, and CMS Day9, group II had lower values than group I, while E Day3, g*_s_* Day3, and g*_s_* Day9 were similar in both groups. Interestingly, C*_i_* Day6, CMS Day6, E Day6, E Day9, g*_s_* Day6, P*_N_* Day3, P*_N_* Day6, P*_N_* Day9, FG, PAN, TIL, and UFG values in group II were higher than those in group I. It appears that more salt-tolerant rice varieties tend to have group II haplotypes than group I haplotypes.

### 2.6. OsCUL3c Gene Expression Based on RNA-Seq Data

**Figure 8:**
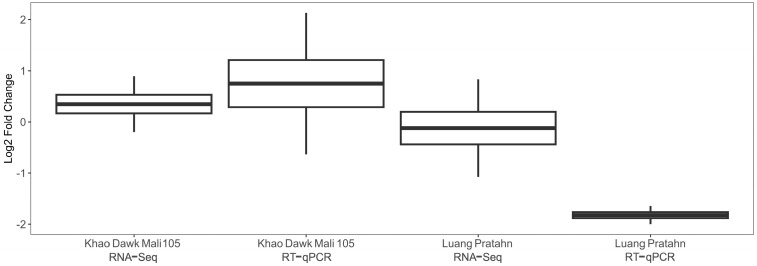
The box plot shows the log2 fold change in differentially expressed *OsCUL3c* genes from RNA-seq data and 2^−ΔΔCt^ values from the RT-qPCR analysis of the representative rice varieties under salt stress conditions. Khao Dawk Mali 105 [25] and Luang Pratahn [26] were used as representatives of the group I and group II haplotypes, respectively.

We were interested in further dissecting the underlying genetic factors that may contribute to the phenotypes under salt stress. We used RNA-seq data from SRA NCBI with unique reads to compare relative changes in the transcriptome profiles of Khao Dawk Mali 105 and Luang Pratahn as representatives possessing group I and group II haplotypes, respectively. To identify differentially expressed genes (DEGs) responsive to salt stress, we performed an analysis between the normal and salt stress data. Our results showed that the *OsCUL3c* gene was upregulated in Khao Dawk Mali 105 and downregulated in Luang Pratahn (Figure 8). The expression level of the DEGs was validated by reverse transcription quantitative PCR (RT-qPCR). The result demonstrated a similar expression pattern as detected in the RNA-seq data analysis. We speculated, based on the box plot, that variations in gene expression of *OsCUL3c* influences the regulation of salt-related responses, and haplotype variations play a role in this regulation.

## 3. Discussion

Three ancestral *cullins* termed *Culα*, *Culβ*, and *Culγ*, appeared in the early evolution of eukaryotes, and evolved after the split of the unikonts (animals and fungi) and bikonts (plants) [27]. Recent findings have identified the *cullins* family in plants, particularly in Arabidopsis [28,29] and rice [14,30]. Several functions of this family have been revealed, including involvement in development, immune signaling, and stress regulation. However, our results clearly show that *cullins* diversity in Arabidopsis and rice has yet to be well annotated considering the information in the four main public repositories, i.e., TAIR, MSU-RGAP, RAP-DB, and NCBI. Four *Cullins* annotated in Arabidopsis can be found in these databases, *AtCUL1*, *AtCUL2*, *AtCUL3*, and *AtCUL4*. In rice, we found three annotated *cullins*, *OsCUL1*, *OsCUL3*, and *OsCUL4*. Still, there are many other *cullins* sequences for which the annotations are unclear or ambiguous. Using a phylogenetic analysis, they were successfully grouped and *CUL3s* were clearly separated from the other *CULs*. This is important because proteins containing a BTB-domain physically interact with CUL3s that are supposed to bridge the substrates for subsequent ubiquitylation [31]. Understanding the function and diversity of the ubiquitylation system is essential to help dissect the pathways in which the system is involved. Therefore, we further examined CUL3 genes in rice and Arabidopsis, which included *OsCUL3a*, *OsCUL3b*, *OsCUL3c* (annotated in this work), *AtCUL3a*, and *AtCUL3b*. The differences among these *CUL3s* were clearly shown by the gene structures and motifs. This work reports the annotation of OsCUL3c, which updates what Lekklar [12] reported on *LOC_Os08g07400* and the annotation reported by Moin [14].

We then investigated the haplotypes in the *OsCUL3c* region using the whole-genome data of 233 local Thai rice accessions and found associations between the haplotypes and salt responses. Over the past two decades, functional genomic studies have demonstrated that the UPS acts as both a positive and negative regulator of the plant stress response. Hereafter, we discussed such roles on the basis of recent studies on the diverse functions of 29 selected Ub ligases, 27 of which are E3 ligases, in abscisic acid (ABA) signaling, dehydration-responsive element binding 2A (DREB2A)-mediated stress tolerance, reactive oxygen species (ROS) homeostasis, and innate immune responses [23]. Through direct or indirect mediation of plant hormones, the UPS selectively degrades key components in stress signaling to either negatively or positively regulate the plant’s response to a given stimulus.

The salt response is a complex trait controlled by genetic and environmental factors. The present study also used coding sequence-, amino acid-, and promoter-based haplotypes to construct phylogenetic trees. The results showed that the six main haplotypes can be categorized into five predominant haplotypes. These predominant haplotypes can be divided into group I, which consists of hap A and hap C, and group II, which consists of hap B1, hap B2, and hap D. Hap B2 contains a small number of accessions (only 10 accessions), so we excluded it from further analysis. The effect of salt stress on the haplotype representatives was examined. The results showed that salt stress had varying effects on the measured parameters. Among the representatives examined, the *OsCUL3c* expression was downregulated in group II and was upregulated in group I, which agreed with the findings of Sonsungsan et al. [26], who reported that Luang Pratahn is more salt-tolerant, and Suratanee et al. [25], who reported that Khao Dawk Mali 105 is less salt-tolerant. Moreover, we also found specific hits for CREs and TFBSs that were particular to both clusters (ABRE*3a*, *ABRE4*, *LTR*, *MYC*, [*AP2*; *ERF*], *NF*-*YB*), which have specific positions and numbers for group I and group II. These elements and factors involved in salt and drought stress and the abscisic acid response might contribute to modulating salt tolerance [32,33,34,35].

**Figure 9:**
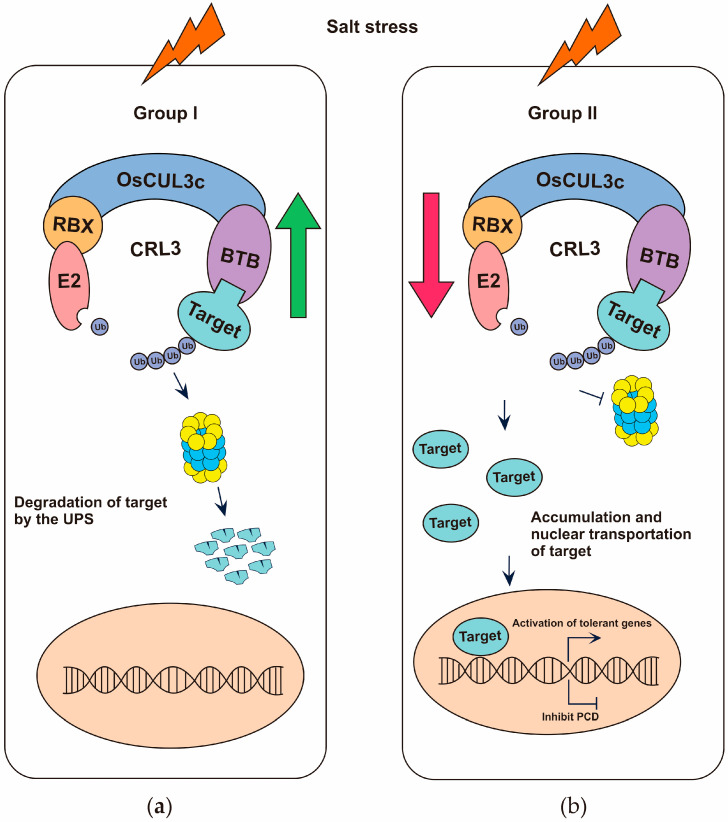
Proposed functional impact of *OsCUL3c* mutations under salt stress. (**a**) Salt stress upregulates *OsCUL3c* expression, resulting in target degradation in group I. (**b**) During salt stress, the *OsCUL3c* target is activated to maintain homeostasis in group II. Green and red arrows depict upregulation and downregulation of *OsCUL3c*, respectively.

This study revealed that *OsCUL3c* haplotypes in local Thai rice accessions have specific associations with the salt stress response. A recent paper indicated that *OsCUL3c* expression levels were increased during salt stress and were dependent on the treatment duration [14]. We investigated the association between these haplotypes, group I and group II, and the salt response. Both haplotypes probably have different expression patterns in response to salt stress, which might be influenced by their different *cis*-regulatory elements and transcription factor binding sites. During salt stress, the increased level of *OsCUL3c* may cause target degradation in group I; on the contrary, in group II, the targets are maintained, which can result in indirect or direct activation of tolerance genes (Figure 9). In addition, amino acid differences between group I and group II haplotypes may affect interactions with their partner, resulting in the degradation of different sets of target proteins. In summary, our investigation of haplotypes associated with salt tolerance supported the role of OsCUL3c in the salt stress response. In addition, the pattern of the haplotype distribution supported the fact that variants of this gene can confer different salt responses, as we observed for *OsCUL3c*, with distinct haplotypes between less salt-tolerant (group I) and more salt-tolerant (group II) rice varieties.

## 4. Materials and Methods

### 4.1. Phylogenetic Construction

All DNA and protein sequences that contained the terms “cullin”, “Oryza sativa” and “Arabidopsis thaliana” were downloaded into a single FASTA file from various public repositories, including the Rice Genome Annotation Project Database (http://rice.uga.edu/, accessed on 24 March 2023), the Rice Annotation Project Database (https://rapdb.dna.affrc.go.jp/, accessed on 24 March 2023), the Arabidopsis Information Resource (https://www.arabidopsis.org/, accessed on 24 March 2023), and the National Center for Biotechnology Information (https://www.ncbi.nlm.nih.gov/, accessed on 24 March 2023). Multiple sequence alignments of all retrieved sequences were carried out using MAFFT 7.511 (https://mafft.cbrc.jp/alignment/software/, accessed on 24 April 2023) using the auto option of $ mafft --auto CUL.fasta > CUL_aln.fasta. The obtained alignments were used for phylogenetic constructions using IQ-TREE 2.0.3 (http://www.iqtree.org/, accessed on 24 April 2023). ModelFinder and a full tree search implemented in the program were used to determine the best-fitting model with a more accurate analysis to reconstruct the corresponding phylogenetic trees with 10,000 replicates of ultrafast bootstrapping; UFBoot was optimized for each bootstrap tree using a nearest-neighbor interchange search. The setting was $ iqtree -s CUL_aln.fasta -m MFP -mtree -B 10000 -bnni. Subsequently, the phylogenetic trees were visualized using iTOL v6 (https://itol.embl.de/, accessed on 25 April 2023). The feature size distribution of the non-redundant CUL3 family was ascertained by identifying gaps in the alignment of coding sequences (CDSs) with genomic sequences using GSDS 2.0 (http://gsds.gao-lab.org/, accessed on 25 April 2023). Their protein sequences were used to predict the conserved motifs using MEME (https://meme-suite.org/meme/tools/meme, accessed on 27 November 2023).

### 4.2. Haplotype Analysis

We created a dataset based on the specific region from the SNPs and indels VCF file [9] using the following command in vcftools (https://github.com/vcftools/vcftools, accessed on 6 June 2023) $ vcftools --vcf input.vcf --chr 8 --from-bp 4153366 --to-bp 4158536 --recode --out OsCUL3c (SNPs or indels, respectively). Both the SNP and indel outputs were then concatenated using a simple command in bcftools (https://github.com/samtools/bcftools, accessed on 6 June 2023): $ bcftools concat --allow-overlaps OsCUL3c.snps.vcf.gz OsCUL3c.indels.vcf.gz --output-type v --output OsCUL3c.snps.indels.vcf. To extract the annotations for chromosome 8, we used a command in awk (https://github.com/onetrueawk/awk, accessed on 10 June 2023) $ awk -F ‘\t’ ‘$1 == “8”’ Oryza_sativa.IRGSP-1.0.55.gff3 > chr8.gff3. The haplotype analysis of the *OsCUL3c* region of the 233 local Thai rice accessions was performed using the GeneHapR 1.1.9 [36] package of R 4.3.0 (https://cran.r-project.org/, accessed on 15 June 2023). To better analyze and draw actionable conclusions, we did not remove haplotypes with heterozygous or missing data. The number of accessions per haplotype was plotted in a bar graph using the ggplot2 [37] package of R. Haplotypes with a minimum of 10 accessions were selected for subsequent analyses. Variants (VCF format) within the coding sequence (CDS) and promoter regions of each selected haplotype were extracted and reconstructed into a FASTA file using a Python script. For the CDS, we used a vcf to fasta command from santiagosnchez (https://github.com/santiagosnchez/vcf2fasta, accessed on 27 June 2023) $ python3 vcf2fasta.py --fasta chr8.fa --vcf hap.vcf.gz --gff chr8.gtf --feat CDS --blend --out cds. The resulting CDSs automatically started from ATG (reverse complement). These sequences were pooled into a single FASTA file and aligned using MAFFT. The CDS-based alignment was colored by variant positions using the following command in alv (https://github.com/arvestad/alv, accessed on 27 June 2023) $ alv -t dna --only-variable cds_aln.fasta. These alignments were translated into their corresponding amino acid (AA) sequences using EMBOSS Transeq (https://www.ebi.ac.uk/Tools/st/emboss_transeq/, accessed on 27 June 2023). The AA-based alignment was colored using the command $ alv -t aa --only-variable aa_aln.fasta. For the promoter, we used the vcf to fasta command from rlebron88 (https://github.com/rlebron88/vcf2fasta, accessed on 3 July 2023) $ python3 vcf2fasta.py --vcf hap.vcf --fasta chr8.fa --output hap.fa. The resulting sequences were still in the same direction as the VCF input; therefore, we generated the reverse complements for these sequences. Furthermore, a file with the promoter sequence was created using the following command in samtools (https://github.com/samtools/samtools, accessed on 5 July 2023) $ samtools faidx hap.fa hap:4157037-4158536 > promoter.fasta. The promoter sequences were then manually curated. The promoter-based alignment was colored using the command $ alv --only-variable promoter_aln.fasta. For all alignments, IQ-TREE and iTOL were used for the phylogenetic tree construction and visualization, respectively.

### 4.3. Cis-Regulatory Elements and Transcription Factor Binding Site Prediction

Putative promoter sequences −1500 bp upstream of the translation start site (TSS) for each haplotype were used in the prediction. Potential cis-regulatory elements (CREs) in the putative promoters were predicted using PlantCARE (https://bioinformatics.psb.ugent.be/webtools/plantcare/html/, accessed on 8 July 2023). For scanning transcription factor binding sites (TFBSs), the putative promoters were queried using PlantPAN 4.0 (http://plantpan.itps.ncku.edu.tw, accessed on 8 July 2023) with *Oryza sativa* as an option. The number of identified CREs and TFBSs was visualized in bar plots using the ggplot2 [37] R package. To visualize the elements and factors positions, the gene structure (reverse) was modeled from *Oryza sativa* Japonica Group (IRGSP-1.0.21). The coordinates of the features were visualized by loading the data frame into R using the genemodel [38] package. Salt and drought (dehydration) stress- and abscisic acid (ABA)-responsive factors positions were visualized using TBtools-II 1.119 (https://github.com/CJ-Chen/TBtools, accessed on 10 July 2023).

### 4.4. Phenotype Analysis

Ten rice accessions, categorized as haplotype A (Khao Gaw Diaw, Khao Dawk Mali 105, Pin Gaew 56, Tah Jeua), haplotype B1 (Khao Luang, Luang Pratahn), haplotype C (Kaset Daw, Dam Dahng, Chai Nat 1), and haplotype D (Khao Gaew), were retrieved based on the availability of phenotypic data from Lekklar [12]. Twenty-one-day-old seedlings were transplanted into a pot containing soil in randomized complete block design with four replicates. At the heading stage, the water on the soil surface was drained and the plants were watered with or without 900 mL of 150 mM NaCl (EC 8−9 dS m^−1^) for 9 days. Salt ions in the soil were washed using tap water until EC < 2 dS m^−1^. The data retrieved included net photosynthetic rate (P*_N_*), stomatal conductance (g*_s_*), transpiration rate (E), and intercellular CO_2_ concentration (C*_i_*), which were measured using a LI-6400 XT portable photosynthesis system (LI-COR, Lincoln, NE, USA). Cell membrane stability (CMS) was measured using a modified method [39]. The numbers of tillers (TILs), panicles (PANs), filled grains (FGs) and unfilled grains (UFGs) were measured after harvesting. The data are expressed as stress susceptibility indices (SSIs), which were calculated by dividing the mean under salt stress by the mean under control conditions [40]. The SSIs were used to draw box plots using the ggplot2 [37] R package.

### 4.5. Gene Expression Analysis

We obtained two published Thai-rice RNA-seq datasets from representatives of group I (Khao Dawk Mali 105; 21 days old treated with 75 mM NaCl) and group II (Luang Pratahn; 14 days old, treated with 115 mM NaCl). Both representatives were recorded at 48 h after the salt stress treatment. The FASTQ files were retrieved from the Sequence Read Archive (SRA) under NCBI project numbers PRJNA507040 [25] and PRJNA747995 [26]. The reads-to-count analysis was conducted on the GALAXY cloud server (https://usegalaxy.eu/, accessed on 10 July 2023). All retrieved files were uploaded to the server as compressed FASTQ files. FastQC 0.11.9 and MultiQC 1.11 were used to visualize the quality at each step. The adapter and low-quality bases were removed using Cutadapt 4.0. Trimmed reads were aligned and mapped to a reference genome (IRGSP-1.0) using Hisat2. From the mapped sequences, the number of reads per annotated genes was counted using FeatureCounts 2.0.0. DESeq2 was used to identify the differentially expressed genes (DEGs). We extracted the *OsCUL3c* log2 fold change data for further analysis. To validate the results obtained from the transcriptomic analysis, the expression level of *OsCUL3c* in the selected representative rice accessions was validated by RT-qPCR. Two varieties, Khao Dawk Mali 105 and Luang Pratahn, were used as representatives of group I and group II, respectively. Two-day-old rice seedlings were transferred into the hydroponic system containing Yoshida solution [41] and grown in a growth chamber (HumanLab, Republic of Korea). After 14 days, the seedlings were treated with and without 100 mM NaCl for 2 days. Leaf tissues were used for RNA extraction using the GENEzol Reagent (Geneaid, Taiwan). The RNA was treated with DNAse I (Thermo Fisher, Waltham, MA, USA) and the first-strand cDNA was synthesized using iScript Reverse Transcription Supermix for RT-qPCR (Bio-Rad, Hercules, CA, USA). Gene-specific primers were designed using Primer3PLUS (http://www.bioinformatics.nl/primer3plus) [42] (Table S3). RT-qPCR was conducted using the Luna Universal qPCR Master Mix (New England Biolabs, Ipswich, MA, USA) and CFX Connect Real-Time PCR Detection System (Bio-Rad, USA), with an initial denaturation at 95 °C for 60 s, followed by 45 cycles of a 2-step PCR: denaturation at 95 °C for 15 s, annealing and extension at 62.8 °C for 30 s. *OsEF-1α* was chosen as an internal reference for data normalization. Five independent biological replicates with three technical replicates of each representative were used. Finally, fold changes in the genes were calculated as mean values of 2^−ΔΔCt^ (Livak) relative to controls and were converted to log2 fold change values. For visual representation, the R package ggplot2 [37] was used to draw the box plot.

## 5. Conclusions

Different transcriptional regulation mechanisms and amino acid substitutions indicated the *OsCUL3c* signature among local Thai rice accessions. Haplotyping revealed that group I (hap A and C) and group II (hap B1 and hap D) were different mutated variants, associated with salt responses. A larger genetic differentiation was observed in the *OsCUL3c* region, providing useful information for future research related to its role in salt stress tolerance and applications in breeding programs.

## Data Availability

The raw transcriptomic data are available in the NCBI BioProject database under accession numbers PRJNA507040 and PRJNA747995. Further detailed data supporting this study’s findings are available from the corresponding author upon reasonable request.

## Supplementary Materials

The following supporting information can be downloaded at: https://www.mdpi.com/article/10.3390/ijms25021040/s1.
