# Supplementary material for: Association of a Specific OsCULLIN3c Haplotype with Salt Stress Responses in Local Thai Rice
Source: Int J Mol Sci. 2024 Jan 15;25(2):1040. doi: 10.3390/ijms25021040 (PMC10815816; doi:10.3390/ijms25021040)
Supplement: Supplementary file 1 [file ijms-25-01040-s001.zip › ijms-2777228-supplementary.pdf]

Supporting Information for: Association of a Specific *OsCULLIN3c* Haplotype with Salt Stress Responses in Local Thai Rice

Bagus Herwibawa, Chakkree Lekklar, Supachitra Chadchawan, and Teerapong Buaboocha

Faculty of Science, Chulalongkorn University, Bangkok 10330, Thailand

E-mail: teerapong.b@chula.ac.th

Supplementary figures and tables

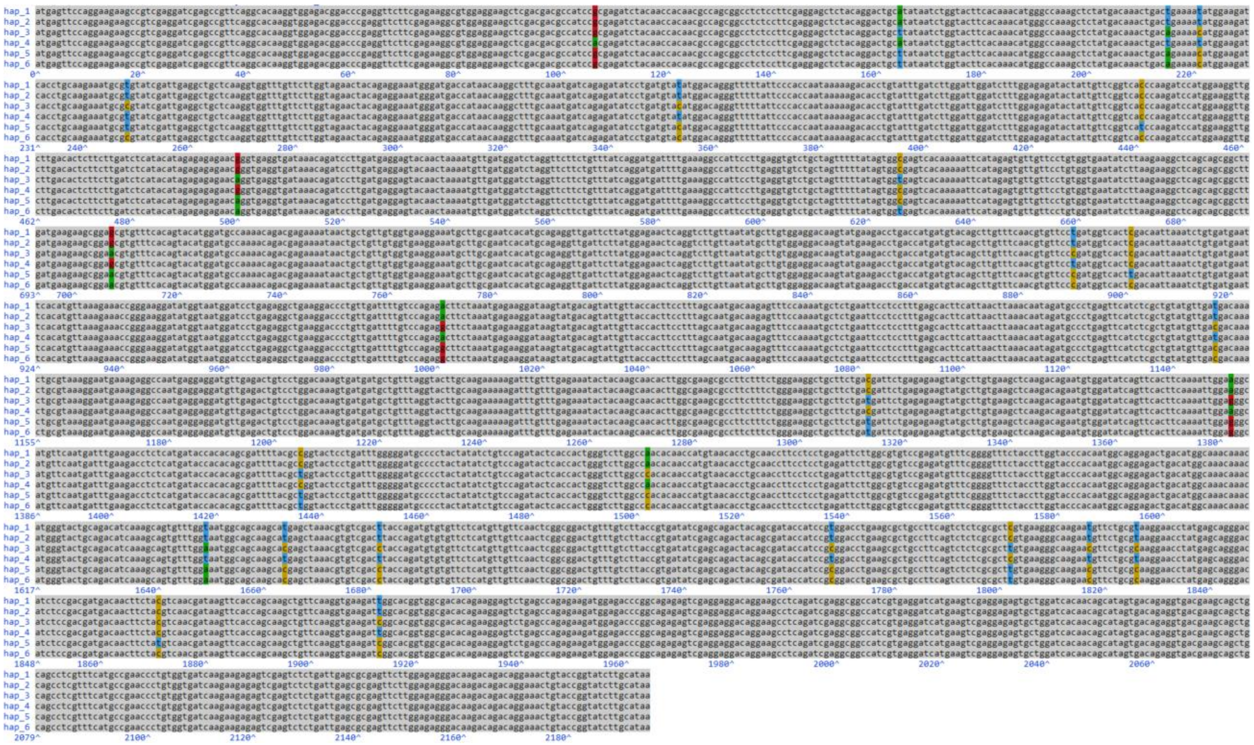

Figure S1. Coding sequence (CDS)-based haplotypes alignment showing conservation of codons. Variant triplet codes/codons are marked with colors.

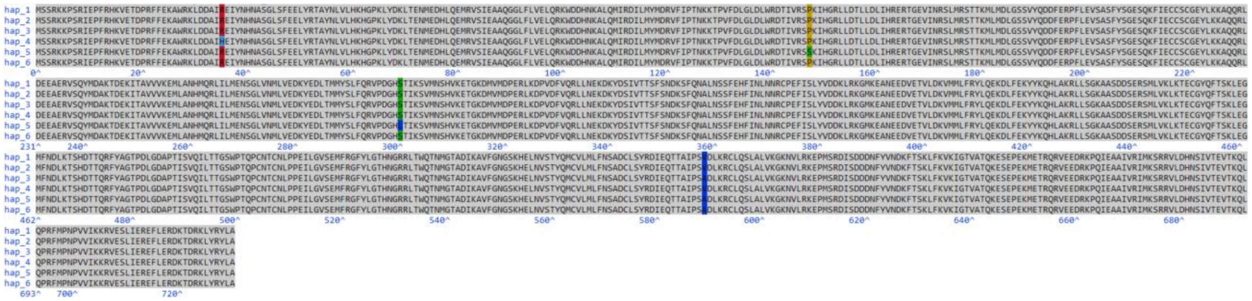

Figure S2. Protein/amino acid (AA)-based haplotypes alignment showing conservation of residues. Variant residues are marked with different colors.

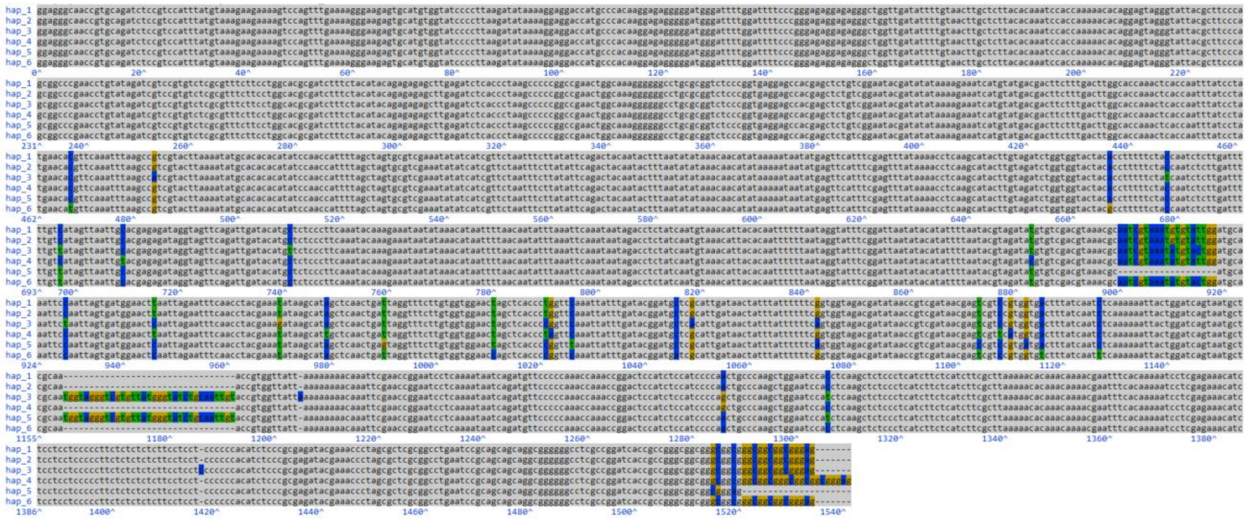

Figure S3. Promoter-based haplotype alignment showing conservation of DNA. Variant DNA are marked with different colors.

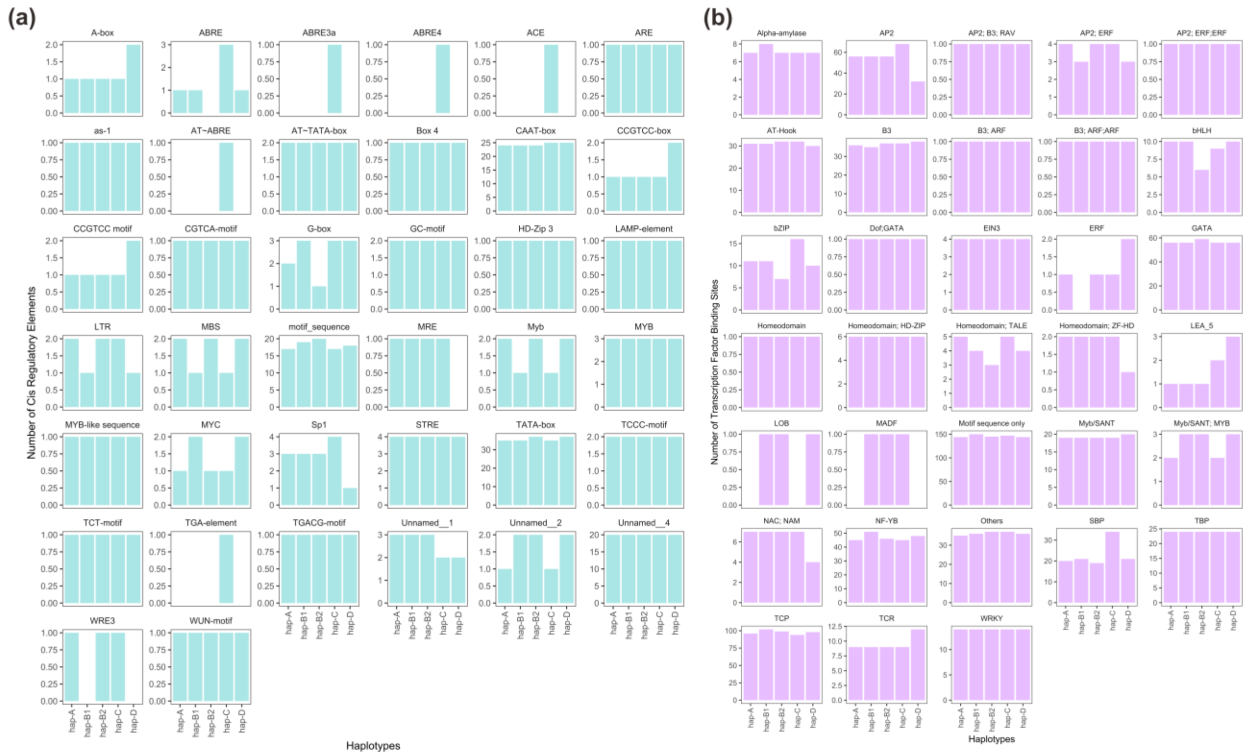

Figure S4. (a) The predicted cis-regulatory elements (CREs) and **(b)** transcription factor binding sites (TFBSs) in the putative promoter sequences of *OsCUL3c*.

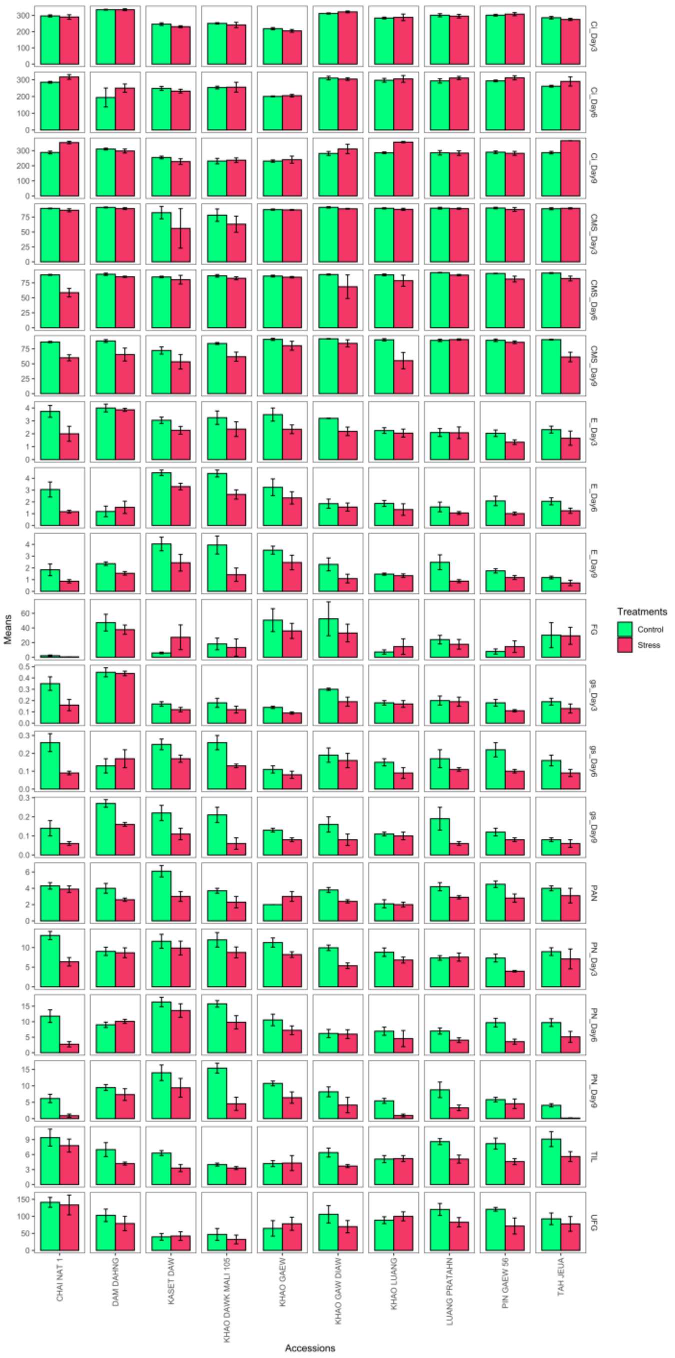

Figure S5. Phenotypes measured in haplotype representatives evaluated under salinity-stress treatment (150 mM NaCl). FG, number of filled grains; PAN, number of panicles; TIL, number of tillers; CMS, cell membrane stability (%); P_N, net photosynthetic rate (μmol CO_2 m^{-2} s^{-1}) ; g_s, stomatal conductance (mol CO_2 m^{-2} S^{-1}); E, transpiration rate (mmol H_2O m^{-2} S^{-1}); Ci, intercellular CO_2 concentration (μmol mol^{-1}) [12].

Table S1 - A set of 233 accessions of local Thai rice used in this study

Acc.	Name of acc.	Acc.	Name of acc.	Acc.	Name of acc.	Acc.	Name of acc.
1	NIC39	73	Khao Supan	145	Leb Nok	217	Gam Liaw
2	Leuang Nuan 807	74	Jed Ruang74	146	Puang Ngern	218	Gam Feuang
3	Tah Mahk 102-10-5	75	Jed Ruang75	147	Lai Maejo	219	Lai Mahk
4	Bahng Pra 166	76	Khao Tah Haeng 17	148	Khao Tah Haeng 17	220	Loi Hah Ruang
5	Khao Nah Prang 126-18E-160	77	Khao Gaew	149	Jed Ruang	221	Chaw Pli Khao
6	Pak Sian 199-33-30	78	Khao Tah Haeng	150	Aew Mod Daeng	222	Niaw Dam Rai
7	Leuang 1-6	79	Khao Gaew	151	Pradoo Daeng	223	Khao' Gam
8	Sai Bua 59-106-2	80	Khao Gaew	152	San-Pah-Tawng Luang	224	Tab Tim Chumpae (RD69)
9	BKN55-11-108	81	Khao Kaset	153	Loei	225	Khao' Hawm Tai Wan
10	BKN57-8-332	82	Wad Boat	154	Khao' Glam	226	RR41
11	Khao Ngah Chahng 107-16-111	83	Rawd Ni	155	Jek Chuey	227	Saeng Lisaw
12	Unknown	84	E-Khawd	156	Alham	228	Ble La
13	Khao Maled Lek	85	Leuang Nawng Kai	157	Nguang Chahng	229	Hawm Supan
14	Leuang Tah Aiam	86	Gahn Mayom	158	Mai Daeng	230	RD43
15	Leuang Rai La Gwian	87	Khao Mana	159	Mali Dam	231	CH1
16	Ta Nod	88	Leuang Sung	160	Mali	232	CH4
17	Tah Khui	89	Setti	161	E-Khiaw Nawn Tung	233	Chai Nat 1
18	Tawng Kam	90	Jed Ruang90	162	Khao' Niaw Dam Maw		
19	Jampah Sawn	91	Jed Ruang91	163	Khao' Niaw Dam Chaw Mai Pai		
20	Leuang Bai Man	92	Khao Luang	164	Khao' Niaw Daeng Grahm Raed		
21	Khao' Kad	93	Jed Ruang	165	Khao' Niaw Dam 4		
22	Hua Kan-nah	94	Unknown	166	Hawm Nahng Nuan		
23	Wad Jan	95	Payah Chom	167	Gam Nai Prom		
24	Gra Pow	96	Khao Tah Haeng96	168	Meuang Nan		
25	Khao' Prae	97	Khao Tah Haeng97	169	Gam Chiang Mai Ton Khiaw		
26	Bai Si	98	Khao Praguad	170	Niaw Dam Hawm Poo Khiaw		
27	Khao Prajuab	99	RD27	171	Daeng Hawm Gulahb		
28	Khao Puang	100	Khao Maled Lek	172	Bahng Taen		
29	Khao Luang	101	Leuang Awn	173	Daw Daeng		
30	Gaew	102	Khao Tah Mon	174	Pra In		
31	Sawng Ruang	103	Pluk Sek	175	Kaset Daw		
32	Khao' Luang Jaek	104	Yai Graeng	176	Nahng Gerd		
33	Khao' Chawad	105	Setti Nawk	177	Daw Yuan		
34	Khiaw Yai	106	Khao Tah Mon	178	Kam Pai		
35	Tawng Mah Eng	107	Payah Chom	179	Lon Lao		
36	Leuang Ton Khaeng	108	Leuang Lah	180	Gled Tow		
37	Leuang Huan	109	Leuang Awn	181	Khitom Luang		
38	Leuang Hahng Mah	110	Pin Tawng	182	Plah Kheng		
39	Ti Nueng	111	Jek Chuey	183	Lawd Gwian		
40	Puang Nahk	112	Khao Tah Jeua	184	E-Meud		
41	Jek Chuey	113	Leuang Roi-et	185	Bua Luang		
42	Khao Gaw Diaw	114	Khao Choo Chahd	186	It Khao		
43	Khao Tah Ex	115	Khwan Chai	187	Khao' Pan		
44	Hawm Surin	116	Khao Tah Jeua	188	Ma Fai Dam		
45	Khao Hah Roi	117	Leuang Roi-et (1)	189	Pa-gah Am-puen		
46	Supanburi 1	118	Leuang Awn Bow	190	Mali Daeng Bow		
47	Pin Gaew	119	Hawm Mali	191	Mali Daeng San Nak		
48	Rawd Ni	120	Maled Lek Bow	192	Mali Daeng Yao Nak		
49	Nok Kao	121	Khao Pahk Grabawk	193	Nahng Dam		
50	Med Ma Kheua	122	Hawm Mali122	194	Sahm Si		
51	Pin Gaew Bow 17-4-27	123	Hawm Mali123	195	Hawm Mali Daeng		
52	OIC5 III	124	Khao Med Lek	196	Khao' Gam		
53	Leuang Awn 151	125	Khao Gaw Diaw	197	Leuang Bahn Sang		
54	Gon Jud 8-4-118	126	Niaw Bai Si	198	Dawk Mai Jan		
55	Ngah Chahng 372	127	Khao Mali	199	Leuang Awn		
56	Klah 199-12-15	128	Khao Dawk Mali 105	200	Khao Nahng Mon		
57	Re Hawm 110-26-77	129	Khao Mali129	201	Pathumthani 1		
58	Khao Bow 466-2-279	130	Khao Mali130	202	Niaw Sew Daeng		
59	BKN55-8-501	131	Khao Tod Lawng	203	Hawm Gra-dang-ngah		
60	Khao Kaset	132	Gow Ruang 17-2-106	204	Gam Nahng Pa-yah		
61	Khua Niaw	133	Pin Gaew 13-24-13	205	Rathu Heenati		
62	Jampah	134	Bahng Pra	206	Hawm Gulahb Daeng		
63	Sabparod	135	Puang Nahk 16	207	Hawm Daeng (Photoperiod insensitive)		
64	Toon Chalawng	136	Khao Jampi 54-8-60	208	Sang Yod		
65	Nahng Bun Mee	137	Nam Dawk Mai 595	209	Hawm Mali Daeng		
66	Ruang Diaw	138	Pin Gaew 56	210	Nok Kum		
67	Gaen Jan	139	Nahng Mon S-4	211	Mae Pa-yah Tawng Dam		
68	Tah Jeua	140	Nahng Chalawng	212	Hawm Nin		
69	Khao Tah Haeng	141	Chaw Ma Gawk Klahng	213	Gam Noi		
70	Khao Tah Haeng	142	Khao Gaw Diaw	214	Mali Dam		
71	Setti	143	Ma Hah Wong	215	Riceberry		
72	Luang Pratahn	144	Niaw Khiaw Ngoo	216	Dam Dahng		

Notes: same name with different accessions number indicates different genomic sequence (GS) number

Table S2 - Haplotype groups and local Thai rice accessions.

Haplotype	Accessions	Frequency
Hap 1	1;10;102;105;113;117;120;125;128;130;131;138;159;167;170;18;189;192;194;196;199;204;210;215;226;23;25;30;44;5;54;63;8	33
Hap 2	112;114;115;118;119;123;129;13;14;142;149;15;151;154;155;179;181;190;195;197;212;213;3;35;36;47;55;60;68;7	30
Hap 3	108;11;110;12;124;135;137;161;2;207;223;29;37;39;4;42;43;48;49;51;61;72;82;86;88;90;93	27
Hap 4	104;111;121;136;147;153;169;175;182;202;205;206;208;21;216;218;233;24;34;38	20
Hap 5	219;70;76;77;78;79;80;81;87;9;96;97	12
Hap 6	162;163;201;225;227;228;230;231;232;46	10
Hap 7	172;69;75;91;92	5
Hap 8	145;156;203;221	4
Hap 9	126;184;186;193	4
Hap 10	144;26;53;64	4
Hap 11	165;178;217;229	4
Hap 12	132;173;31;94	4
Hap 13	116;122;65;99	4
Hap 14	134;171;32	3
Hap 15	183;22;56	3
Hap 16	139;211	2
Hap 17	198;45	2
Hap 18	222	1
Hap 19	101	1
Hap 20	83	1
Hap 21	74	1
Hap 22	103	1
Hap 23	84	1
Hap 24	100	1
Hap 25	95	1
Hap 26	164	1
Hap 27	150	1
Hap 28	148	1
Hap 29	89	1
Hap 30	59	1
Hap 31	71	1
Hap 32	200	1
Hap 33	188	1
Hap 34	166	1
Hap 35	187	1
Hap 36	20	1
Hap 37	98	1
Hap 38	66	1
Hap 39	107	1
Hap 40	6	1
Hap 41	185	1
Hap 42	17	1
Hap 43	141	1
Hap 44	73	1
Hap 45	214	1
Hap 46	41	1
Hap 47	152	1
Hap 48	143	1
Hap 49	57	1
Hap 50	177	1
Hap 51	168	1
Hap 52	174	1
Hap 53	109	1
Hap 54	224	1
Hap 55	62	1
Hap 56	19	1
Hap 57	160	1
Hap 58	157	1
Hap 59	67	1
Hap 60	27	1
Hap 61	28	1
Hap 62	180	1
Hap 63	33	1
Hap 64	16	1
Hap 65	40	1
Hap 66	127	1
Hap 67	140	1
Hap 68	106	1
Hap 69	50	1
Hap 70	209	1
Hap 71	146	1
Hap 72	85	1
Hap 73	133	1
Hap 74	220	1
Hap 75	191	1
Hap 76	158	1
Hap 77	58	1
Hap 78	52	1
Hap 79	176	1

Table S3 - Primers used for RT-qPCR validation

Primer name	Type	Primer sequence (5′ -> 3′)	Tm (ºC)	Product size (bp)
RT_OsCUL3_F	Forward	ACCAGCAAGCTGTTCAAGGT	57.1	188
RT_OsCUL3_R	Reverse	TGCTTCGTCACCTCTGTCAC	57.1	
OsEF-1α _F	Forward	ATGGTTGTGGAGACCTTC	53.7	127
OsEF-1α _R	Reverse	TCACCTTGGCACCGGTTG	58.2	
